# Effect of Mitomycin C on Myopic versus Astigmatic Photorefractive Keratectomy

**DOI:** 10.1155/2017/2841408

**Published:** 2017-03-14

**Authors:** Ashwag A. Almosa, Samah M. Fawzy

**Affiliations:** ^1^College of Applied Medical Science, King Saud University, P.O. Box 2454, Riyadh 11451, Saudi Arabia; ^2^Faculty of Medicine, Ain Shams University, Ramses St., Abbasia, Cairo 191566, Egypt

## Abstract

*Purpose*. Long-term mitomycin C (MMC) effects on photorefractive keratectomy (PRK) were compared in simple myopic and astigmatic patients*. Methods*. In this observational cohort study, subjects were selected based on preoperative and postoperative data collected from medical records; they were divided into simple myopia with/without MMC and myopic astigmatism with/without MMC groups. Haze, uncorrected visual acuity (UCVA), best-corrected visual acuity (BCVA), subjective refraction, and K-reading were evaluated at 1-, 3-, 6-, and 12-month follow-ups. *Results*. One hundred fifty-nine eyes of 80 subjects (34 women and 46 men; mean age, 26.81 ± 7.74 years; range, 18–53 years; spherical powers, −0.50 to −8.00 DS; and cylindrical powers, −0.25 to −5.00 DC) were enrolled. One year postoperatively, the simple myopia with/without MMC groups showed no difference in UCVA (*P* = 0.187), BCVA (*P* = 0.163), or spherical equivalent (*P* = 0.163) and a significant difference (*P* = 0.0495) in K-reading; the haze formation difference was nonsignificant (*P* = 0.056). Astigmatic groups with/without MMC showed a significant difference in K-reading (*P* < 0.0001). MMC groups had less haze formation (*P* < 0.0001). *Conclusion*. PRK with intraoperative MMC application showed excellent visual outcomes. MMC's effect on astigmatic patients was significantly better with acceptable safety and minimal side effects.

## 1. Introduction

In 1975, Stuart Searles produced the first excimer laser [1]. Trokel was the first to show that the excimer laser is able to remove corneal tissue precisely without any damage to adjacent tissues. The first human eye underwent refractive surgery in 1988 and was treated by McDonald and coworkers [2, 3]. The excimer laser is used in three major refractive surgeries: photorefractive keratectomy (PRK), laser epithelial keratomileusis (LASEK), and laser-assisted in situ keratomileusis (LASIK) [2].

PRK is a successful surgical treatment for refractive errors. The main drawback of PRK or any surface ablation is postoperative haze formation [4]. Severe and dense haze is treated with the use of a topical corticosteroid, but that results in more complications with long-term use such as increased intraocular pressure (IOP), glaucoma, and cataract formation [5]. Nowadays, mitomycin C (MMC) is widely used to prevent postablation haze. MMC 0.02% is an antineoplastic antibiotic that selectively inhibits the syntheses of DNA, RNA, and proteins. It is used intraoperatively after ablation; in the more common technique, MMC is applied or wiped on top of the stroma after the ablation, and recent studies suggest that the application time can vary according to ablation depth and refractive error [2]. The efficacy and predictability of PRK with the intraoperative application of MMC have already been reported in several studies. Most concentrated on haze prevention in certain types of refractive error. In our study, we investigated the effects of MMC on highly myopic patients and in highly astigmatic patients, as these are the main concerns in all surface ablation surgeries.

## 2. Materials and Methods

### 2.1. Ethical Approval

All research conducted adhered to the tenets of the Declaration of Helsinki (1964), and ethical approval to conduct this study was obtained from Eye World Medical and Surgical Complex. Moreover, an informed consent form was obtained from patients after they received information on the objective and methodology of the study.

### 2.2. Study Subjects

This observational cohort study was conducted at Eye World Medical and Surgical Complex in Riyadh City, Kingdom of Saudi Arabia, between March 2014 and May 2015. Subjects were selected based on preoperative and postoperative data collected from medical records. One year postoperatively, they were recalled to fulfill the last stage of the evaluation, which was corneal topography. The selected preoperative data were obtained from patients aged 18 years and older. The patient's spherical power must be in the range from −0.50 DS to −8.00 DS; for patients having compound myopic astigmatism, the cylindrical power must be in the range from −0.25 DC to −5.00 DC with a best-corrected visual acuity (BCVA) of 20/25 or better. For contact lens users, only those who had stopped wearing contact lenses 2 weeks before surgery were selected. The patients must be free from any systemic or ocular conditions and generally healthy. The patients were divided according to their refractive errors and the use of MMC during surgery into four groups: simple myopia without MMC, simple myopia with MMC, compound myopic astigmatism with MMC, and compound myopic astigmatism without MMC.

### 2.3. Study Examinations and Procedures

The preoperative data included uncorrected visual acuity (UCVA) and BCVA (RT 2100; NIDEK Co. Ltd., Gamagori, Japan), subjective refraction, slit-lamp examination (AIA-12 Zoom; APPASAMY Co. Ltd., Tamil Nadu, India), fundus examination with indirect ophthalmoscopy, corneal topography (Allegro Oculyzer; WaveLight Technologie AG, Erlangen, Germany), pupil diameter (scotopic and photopic) (Auto Kerato-Refractometer KR-8800; Topcon Co. Ltd., Tokyo, Japan), IOP (TOPCON CT-80 Non-Contact; Topcon Co. Ltd.), and Schirmer's test for dryness.

The excimer laser used in this study was the 400 Hz Allegretto Wave Eye-Q (WaveLight Technologie AG). For the groups of patients treated with MMC, a concentration of 0.02 mg/mL was applied at that time, and the duration of application varied according to ablation depth: the greater the ablation depth, the longer the application. The duration of application ranged from a minimum of 15 seconds to a maximum of 30 seconds.

All patients received the same medications postoperatively and had the same treatment plan, which was gatifloxacin ophthalmic solution 0.3% (ZYMAR, Allergan, Weston, FL, USA) and prednisolone acetate ophthalmic suspension USP 1% (PRED FORTE, Allergan) for 1 week postoperatively, lubricant eye drops (REFRESH TEARS, Allergan) for 3 weeks postoperatively, and fluorometholone ophthalmic solution (FML, Allergan) for 6 weeks postoperatively starting from the fourth postoperative week as a replacement for prednisolone acetate for 3 months.

Data were recorded at follow-up visits scheduled 1, 3, and 6 months postoperatively; a full slit-lamp examination, visual acuity testing, and subjective refraction testing were performed. One year postoperatively, the patients were recalled to fulfill the last step of the research where the same routine follow-up examination was performed in addition to corneal topography by an Allegro Oculyzer (WaveLight Technologie AG, Erlangen, Germany).

### 2.4. Data Analyses

A paired *t*-test (GraphPad version 3.1 for Windows, GraphPad Software, La Jolla, CA, USA) was used to obtain *P* values between simple myopic patients with and without MMC and astigmatic patients with and without MMC at each follow-up visit. *P* values < 0.05 were considered significant. Moreover, an analysis of variance (ANOVA) was performed between all four groups.

## 3. Results

A total of 159 eyes of 80 patients (34 women and 46 men; mean age, 26.81 ± 7.74 years; range, 18–53 years) were treated with PRK. The mean spherical refractive error in the myopic groups was −4.08 ± 2.17 DS (range, −0.5 to −8.5 DS), while that in the astigmatic groups was −3.65 ± 2.69 DS (range, −0.5 to −8.5 DS). The mean cylinder power of the astigmatic group was −1.72 ± 1.05 DC (range, −0.25 to −4.25 DC). In this study, we compared the effect of intraoperative MMC on the correction of simple myopia and astigmatism. To observe this effect, the following parameters were compared: UCVA, BCVA, refractive errors, haze formation, and K-reading values.

All four groups underwent the same follow-up protocol and statistical analysis at each visit 1, 3, 6, and 12 months postoperatively. We wanted to know the effect of MMC on the simple myopia and astigmatism groups in the long term. One year postoperatively, a comparison of simple myopia with MMC use and simple myopia without MMC use was performed and demonstrated that the K-reading value tended to be flatter in the MMC group (39.23 ± 2.1) compared with the non-MMC group (40.58 ± 1.23) (*P* = 0.0495). However, for the other parameters, there was no clinically or statistically significant difference between the two groups. There was no statistically or clinically significant difference in refractive errors between the groups with or without MMC (*P* = 0.1631).

In the astigmatism group, when we compared the parameters 1 year postoperatively, there was a clinically and statistically significant difference between the two groups in K-reading values; the MMC groups had flatter keratometry readings (39.33 ± 1.7) compared with the non-MMC groups (40.98 ± 1.35) (*P* < 0.0001).

**Figure 1:**
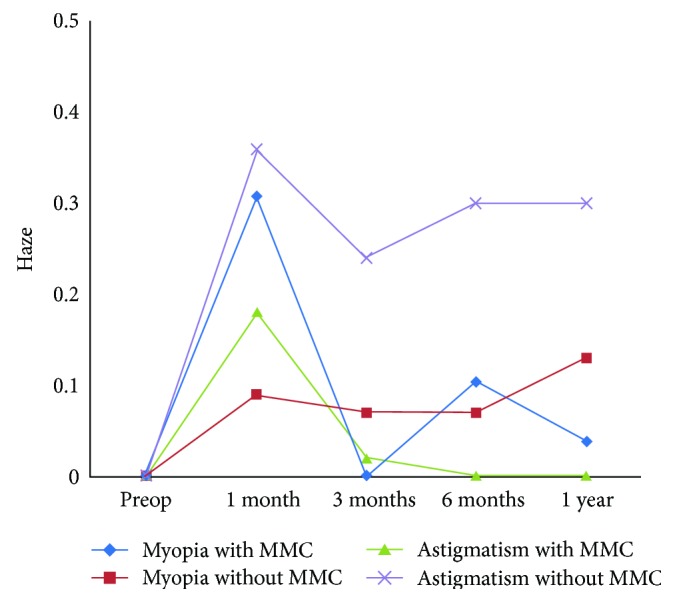
Haze progression over time in all four groups.

The refractive power in astigmatic subjects showed no statistically significant difference between the MMC and non-MMC groups (*P* = 0.0613). The effect of MMC was observed in haze formation, as the MMC groups tended to have lower haze formation with a mean of zero compared with the non-MMC groups (0.31 ± 0.32) (*P* < 0.0001) (Table 1). The haze peaked 1 month postoperatively in all four groups and then reached its lowest point 3 months postoperatively. Then, the non-MMC groups started to show more haze at 1 year, especially in the astigmatic group (Figure 1 and Table 2).

## 4. Discussion

MMC (0.02 mg/mL) is used commonly nowadays during PRK to reduce haze formation after surgery. Some of the main concerns with its use are its concentration and the duration of application. In the current study, we used MMC 0.02%, as this is the safest and most efficient concentration with no adverse reaction [6]. Recent studies suggest that the duration of application depends on the amount of ablation; the higher the refractive error, the longer the application time, which ranges from 15 to 120 seconds [6]. This is the procedure we followed in this study when applying MMC.

In the current study, we compared the effect of MMC between simple myopic and compound myopic astigmatism. Haze formation was statistically analyzed in the myopic and astigmatic groups who underwent surgery with and without the use of MMC, and there were clinically and statistically significant differences in haze formation (*P* < 0.0001) that peaked 1 month postoperatively in the four groups. Then, the haze started to diminish and reached its lowest mean value 3 months postoperatively. The highest mean value for haze was found among astigmatic patients without MMC. Our findings show that the use of MMC is important to minimize haze formation postoperatively and to maintain good visual outcomes, especially in astigmatic patients with higher cylinder powers, which agree with the report from the American Academy of Ophthalmology on MMC in corneal surface excimer laser ablation techniques [7, 8]. MMC is used in PRK to inhibit subepithelial fibrosis as a result of abnormal proliferation of stromal keratocytes after ablation [2].

Similar results were found by Bedei et al. in 2006 [9]. Moreover, Gambato et al. in 2005 [10] performed a clinical trial on 36 highly myopic patients who underwent PRK with MMC on one eye and PRK with artificial tears on the fellow eye and reported corneal haze in 20% of the controls versus 0% in MMC-treated eyes after 1 year. In addition, Thornton et al. in 2007 [11] compared the effect of low-dose MMC 0.002% versus no MMC on haze values. They found significantly less haze in MMC eyes at all postoperative follow-up visits with a mean peak haze of 1.4 at 2 months for the non-MMC eyes and a mean peak haze of 0.5 at 1 month for the MMC-treated eyes. In 2016, Hashemi et al. [12] evaluated the results of PRK with MMC in myopia correction after 5 years (mean spherical equivalent before surgery, −3.40 ± 1.73), and they found no haze formation and good visual stability 5 years after surgery.

In contrast, Hofmeister et al. [5] found a significant difference in haze scores between MMC-treated eyes and untreated eyes at 1 and 3 months (*P* = 0.034) but no difference at 6 or 12 months.

In the current study, K-reading values were statistically analyzed in all four groups and were affected by MMC use; values seemed to be flatter among MMC patients with myopia and astigmatism compared with non-MMC patients with myopia and astigmatism. To explain this finding, our assumption is that with MMC, the corneal stroma had less keratocyte proliferation and thus a decreased density of keratocytes; hence, the deposition of new corneal collagen fibers occurs. This may lead to a flatter curvature of the cornea. No other study has compared the effect of MMC on corneal curvature post-PRK. This finding needs further investigation, as the curvature of the cornea plays an important role in vision stabilization, and we do not want a very flat cornea postoperatively when using MMC, especially with high refractive errors and thinner corneas.

The current study showed no clinical or statistical difference among the four groups in either UCVA or BCVA. The same result was found by Thornton et al. [6] in 2008 when they compared the effect of low-dose MMC 0.002% versus no MMC on myopic patients. However, Carones et al. [13] in 2002 noted better UCVA and BCVA and more accurate refractive outcomes with prophylactic use of a single dose of MMC 0.02% at the end of PRK surgery as compared with the other groups. This difference might be attributed to the fact that in their non-MMC-treated group, a higher grade of haze was noted that caused visual acuity decline, while in our study, no patient in either group had a haze grade of more than 1 and that is why the UCVA and BCVA were not affected and showed no difference.

Some patients had residual refractive errors that peaked 1 month postoperatively. The ANOVA showed neither clinical nor statistical difference among any of the four groups (*P* = 0.3840). Vision then started to stabilize 1 year postoperatively. Another study on 124 eyes of 62 patients by Bedei et al. in 2006 [9] reported similar results. Ghoreishi et al. [14] in their 2009 study found that the spherical equivalent 1 year postoperatively with MMC was within ±0.50 D in 69.4% and within ±1.00 D in 91% of the eyes. However, in the present study, no patient showed any considerable residual refractive error 1 year postoperatively. The difference may be attributed to the range of refractive errors (−2.50 to −13.5 DS) in their study being higher than the range in our study (−0.50 to −8.50 DS) and differences in surgical procedure techniques; in the current study, we used the FCAT technique with the *Q* value encoded in the excimer laser machine.

## 5. Conclusions

PRK with intraoperative application of MMC showed excellent visual outcomes. Moreover, the predictability of long-term stabilization was better with the use of MMC. The effect of MMC on astigmatic patients was significantly better with acceptable safety and minimal side effects.

Although this study reached its aim, there were some limitations that could not be avoided. There was a short period of time to collect the data, and patients who did not comply with all follow-up visits reduced the sample size. Moreover, patients did not undergo a contrast sensitivity test preoperatively, and this meant we could not compare it as a parameter in the study. We suggest making the contrast sensitivity test routine in refractive surgery work-ups.

We recommend further investigations with longer follow-up periods, especially for K-reading postoperatively with MMC use. Additionally, more investigation is needed on endothelial cell defects and counts after MMC use. More research is needed on the effect of MMC on residual astigmatism following PRK.

In summary, the use of MMC in astigmatic patients reduced haze formation significantly more than that in myopic patients.

## Conflicts of Interest

The authors declare that there is no conflict of interest regarding the publication of this paper.

## Figures and Tables

**Table 1:** A 1-year postoperative comparison of patients with simple myopia with/without MMC and astigmatic patients with/without MMC. An ANOVA test compared the four groups.

Variable	Simple myopia		*P* value	Astigmatism		*P* value	ANOVA (four groups)
	MMC	No MMC	Paired *t*-test	MMC	No MMC	Paired *t*-test	
UCVA (in logMAR)	0 ± 0	0.1 ± 0.141	*P* = 0.1872	0.04 ± 0.137	0 ± 0	*P* = 0.1872	0.4624
BCVA (in logMAR)	0 ± 0	0.05 ± 0.070	*P* = 0.1631	0.04 ± 0.137	0 ± 0	*P* = 0.1703	0.5193
Refractive error	0	−0.03 ± 0.08	*P* = 0.1631	−0.027 ± 0.098	−0.016 ± 0.056	*P* = 0.0613	0.4798
K-reading	39.23 ± 2.1	40.58 ± 1.23	*P* = 0.0495	39.33 ± 1.7	40.98 ± 1.35	*P* < 0.0001	0.0049
Haze formation	0	0.14 ± 0.287	*P* = 0.0560	0	0.31 ± 0.32	*P* < 0.0001	<0.0001

ANOVA: analysis of variance; BCVA: best-corrected visual acuity; MMC: mitomycin C; UCVA: uncorrected visual acuity.

**Table 2:** A 1-year postoperative comparison between the MMC and non-MMC groups.

	With MMC		*P* value	Without MMC		*P* value
	Simple myopia	Astigmatism		Simple myopia	Astigmatism	
UCVA	0 ± 0	0.036 ± 0.137	*P* = 0.1700	0.1 ± 0.141	0 ± 0	*P* = 0.1872
BCVA	0 ± 0	0.036 ± 0.137	*P* = 0.1700	0.05 ± 0.070	0 ± 0	*P* = 0.1631
Refractive error	0 ± 0	0.004 ± 0.102	*P* = 0.7643	−0.028 ± 0.08	−0.016 ± 0.056	*P* = 0.3313
K-reading	39.23 ± 2.1	39.33 ± 1.7	*P* = 0.3995	40.58 ± 1.230	40.98 ± 1.355	*P* = 0.0625
Haze formation	0	0	No difference	0.14 ± 0.287	0.31 ± 0.32	*P* = 0.0204

BCVA: best-corrected visual acuity; K-reading: keratometry reading; MMC: mitomycin C; UCVA: uncorrected visual acuity.
